# A Comparison of Bonded and Nonbonded Zinc(II) Force Fields with NMR Data

**DOI:** 10.3390/ijms24065440

**Published:** 2023-03-13

**Authors:** Milana Bazayeva, Andrea Giachetti, Marco Pagliai, Antonio Rosato

**Affiliations:** 1 Magnetic Resonance Center (CERM), University of Florence, Via Luigi Sacconi 6, 50019 Sesto Fiorentino, Italy; 2 Department of Chemistry, University of Florence, Via della Lastruccia 3, 50019 Sesto Fiorentino, Italy; 3 Consorzio Interuniversitario di Risonanze Magnetiche di Metallo Proteine, Via Luigi Sacconi 6, 50019 Sesto Fiorentino, Italy

**Keywords:** zinc, zinc-finger, NMR, order parameter, heteronuclear NOE, molecular dynamics, AMBER, ZAFF, metals, metalloprotein

## Abstract

Classical molecular dynamics (MD) simulations are widely used to inspect the behavior of zinc(II)-proteins at the atomic level, hence the need to properly model the zinc(II) ion and the interaction with its ligands. Different approaches have been developed to represent zinc(II) sites, with the bonded and nonbonded models being the most used. In the present work, we tested the well-known zinc AMBER force field (ZAFF) and a recently developed nonbonded force field (NBFF) to assess how accurately they reproduce the dynamic behavior of zinc(II)-proteins. For this, we selected as benchmark six zinc-fingers. This superfamily is extremely heterogenous in terms of architecture, binding mode, function, and reactivity. From repeated MD simulations, we computed the order parameter (S^2^) of all backbone N-H bond vectors in each system. These data were superimposed to heteronuclear Overhauser effect measurements taken by NMR spectroscopy. This provides a quantitative estimate of the accuracy of the FFs in reproducing protein dynamics, leveraging the information about the protein backbone mobility contained in the NMR data. The correlation between the MD-computed S^2^ and the experimental data indicated that both tested FFs reproduce well the dynamic behavior of zinc(II)-proteins, with comparable accuracy. Thus, along with ZAFF, NBFF represents a useful tool to simulate metalloproteins with the advantage of being extensible to diverse systems such as those bearing dinuclear metal sites.

## 1. Introduction

Zinc is an essential element for all cells [1]. It is the second most abundant transition metal ion in living organisms after iron. Zinc(II)-binding proteins are key players in an extensive variety of biochemical processes such as protein synthesis and degradation, DNA metabolism and repair, and neurotransmission [2,3]. In order to obtain a detailed understanding at the atomic level of the mechanisms by which zinc(II)-binding proteins carry out their function, it is important to have information on their 3D structures and on their dynamics properties. The former is typically obtained through structural biology methods, such as X-ray diffraction, NMR spectroscopy or cryo-EM. The information on dynamics is more difficult to probe experimentally in a direct manner, NMR spectroscopy being the most apt technique to this end [4,5]. Alternatively, classical molecular dynamics (MD) simulations provide a powerful tool to understand how metal binding impacts the behavior of a protein in solution, at both the structural and dynamics level [6,7,8]. The reliability of simulations is related to the availability of an accurate force field (FF) [9].

Different models have been developed to parameterize metals in biological systems and their protein ligands (i.e., the protein residues containing the atoms that interact directly with the metal ion via the coordination bond) [10,11]. The two major approaches are the bonded and nonbonded models. The former incorporates explicitly the coordination bonds between the metal and the donor atoms of the protein as well as of any other molecule (e.g., inhibitor) interacting directly with the metal. The coordination bond is represented via bond and angle terms, while the torsion term is usually neglected. The charges are often computed using the RESP method for the metal cation and for the ligands [12]. One main disadvantage of such a model is that it entails a burdensome parametrization for each specific system under study. In this work, we used the well-known and extensively used zinc AMBER force field (ZAFF) [13]. On the other hand, the nonbonded approach treats the metal ion as a sphere with appropriate electrostatics and van der Waals (vdW) terms to describe the interaction with the ligands. This strategy reflects the nature of the zinc(II) interactions in the binding sites, permitting transient modifications of the coordination geometry or exchanges with the solvent or nearby protein residues. Moreover, this model is convenient in terms of computational speed [14,15]. Thus, as an alternative to the aforementioned ZAFF model, we used a nonbonded parametrization of zinc(II) that was developed by some of us relatively recently. Two additional approaches are available in the literature, namely the cationic dummy model and the polarizable model. In the cationic dummy model, the metal is covalently bound to dummy particles with a defined geometry. Because there are no bonds between the dummy sites and the ligands, this rigid complex can move freely around its frame, change coordination geometry and exchange ligands. The charge of the metal is distributed over the entire complex, to reflect the partially covalent nature of the coordination bonds [10]. Finally, the polarizable model aims to reproduce the charge delocalization as a function of the coordination environment [16,17,18]. However, the polarizable approach is expensive from a computational point of view and hence it is seldom used in MD simulations [10,11].

The aim of this work was to assess the bonded and nonbonded models for the parametrization of zinc(II) sites with respect to their capability to provide accurate information on the dynamics of zinc(II)-binding proteins. Both models are not particularly demanding in terms of computational cost and differ mainly because the nonbonded approach is more easily portable to a variety of different systems and it is suitable to model ligand exchanges in the metal coordination sphere, while it may result in less stable MD trajectories due to e.g. the metal detaching from the protein. A strategy already adopted for the validation of FFs in proteins that do not harbor metal cofactors is to compare experimental NMR observables with predictions obtained from the simulations [19]. For the present investigation we focused on ^1^H-^15^N nuclear Overhauser effect (Het-NOE) data [20] measured for zinc-finger proteins. Het-NOE data are good reporters of protein backbone mobility on the sub-ns time scale, which can be sampled very well by classic MD simulations. Our results suggest that both models are well suited to reproduce the experimentally observed dynamics over the entire protein. In particular, there are no significant differences between the models even for the dynamics of the protein residues within the zinc(II) binding sites.

## 2. Results

### 2.1. Background

**Figure 1:**
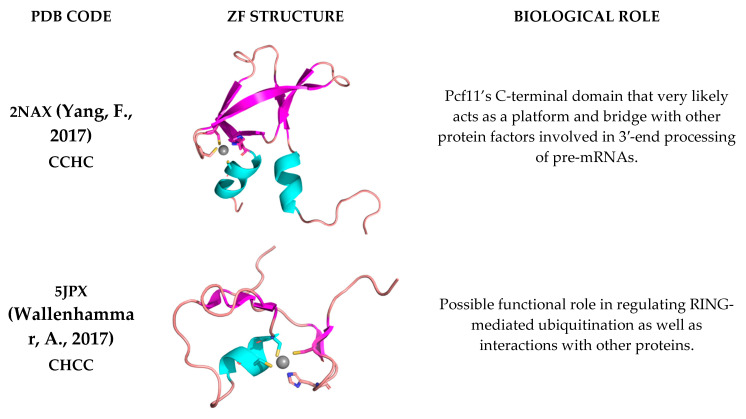

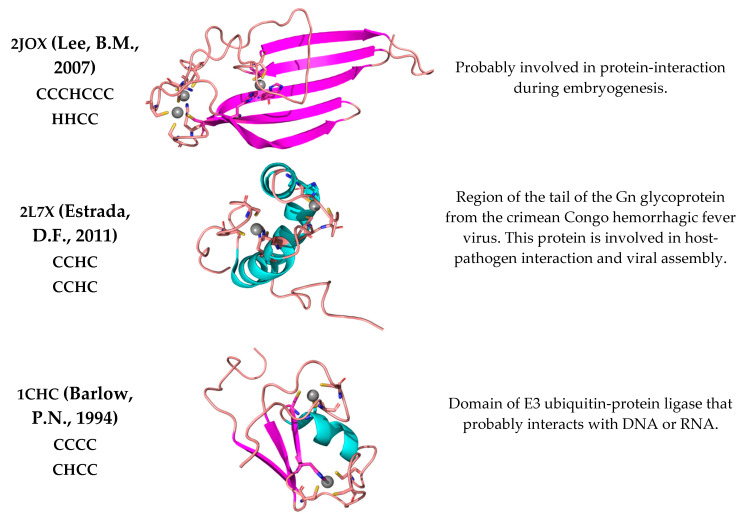

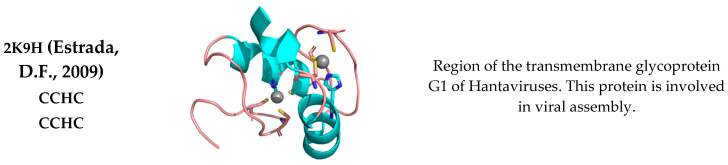
Zinc-fingers used to test the bonded and nonbonded FF. The first column reports the PDB code of the structure together with the amino acid pattern(s) binding the zinc(II) ion(s) [23,24,25,26,27,28,37,38]. The third column reports information about their biological role.

In this work, we focused on zinc-fingers (ZFs), which are among the most structurally diverse metalloprotein domains. They present various architectures, metal binding modes, functions, and reactivity [21,22]. Here, we selected NMR structures (PDB codes: 2NAX, 5JPX, 2JOX, and 2L7X) of ZFs that were characterized also through heteronuclear nuclear Overhauser effect (Het-NOE) measurements [23,24,25,26]. To further expand the structural diversity of our dataset, i.e., target different protein topologies, we included two additional ZF structures (PDB codes: 1CHC, 2K9H), for which unfortunately there are no relaxation data available [27,28]. Our benchmark structures contained one or two independent zinc(II) sites as well as, in one case, a binuclear site (Figure 1).

In this study, we aimed to evaluate two different FFs for zinc(II). The agreement with experimental data is a reliable measure of the accuracy of the FFs [9,19,29,30]. NMR spectroscopy is used to obtain information about protein motions on a broad range of timescales, as nuclear spin relaxation rate reports on the internal motions on different timescales as well as on the overall rotational diffusion of the molecule. The three commonly measured NMR relaxation rates are the spin-lattice relaxation rate (R_1_), the spin-spin relaxation rate (R_2_), and Het-NOE data for all the ^1^H-^15^N moieties in the protein. Het-NOE data are extremely sensitive to fast protein dynamics [31,32,33]. Since all amino acids except Pro contain at least one N-H moiety within the peptide bond, these data provide a comprehensive coverage of the flexibility of the entire protein chain.

A core assumption of most strategies to interpret NMR relaxation data in proteins is the decoupling of the overall and the internal motions. Information about local motions is derived by fitting suitable parametric functions to the relaxation rates, e.g., as done in the so-called model-free approach [31,34,35]. The latter is termed model-free because the parameters are derived without the need to invoke a specific model for the internal motion. The model-free analysis of the data mentioned in the previous paragraph outputs a set of parameters for each N-H bond in the protein. In particular, the order parameter (S^2^) describes the magnitude of the angular fluctuation of each bond vector, reflecting the flexibility of the polypeptide at those sites with respect to the overall frame [31,34]. However, we chose to compare the MD-derived S^2^ with the Het-NOE data rather than the NMR-derived S^2^ values, because Het-NOEs are experimental data that can be used without any interpretation or assumption and report on the relevant timescale of dynamics (sub-ns).

### 2.2. Analysis of the MD Simulations and Comparison of Simulated vs. Experimental Dynamics

The overall protein fold remained stable during the production phase for all systems, as shown by the RMSD values of the backbone (Supplementary Figures S1–S6). When using the NBFF, the electrostatic nature of the coordination allows transient distortions of the zinc(II) site and may lead, in principle, to the protein losing its metal cofactor [10,14]. For this reason, we inspected the donor atom–metal distances throughout the trajectories. The zinc(II) coordination was maintained during all MD runs, with fluctuations of 0.04 Å around the equilibrium distances. This behavior agrees with previous reports for other systems [14,36]. Instead, in the bonded simulations, the metal was kept fixed to the donor atoms through covalent bonds [13], so there was no need to monitor these distances. These data indicated that all MD runs, with both FFs, were suitable for our subsequent analyses. As mentioned in the preceding section, we used these trajectories to compute the S^2^ parameters. In turn, this information allowed us to assess whether there were differences in the accuracy of the protein dynamics simulated with the two FFs based on the comparison with the experimental NMR observables.

**Figure 2:**
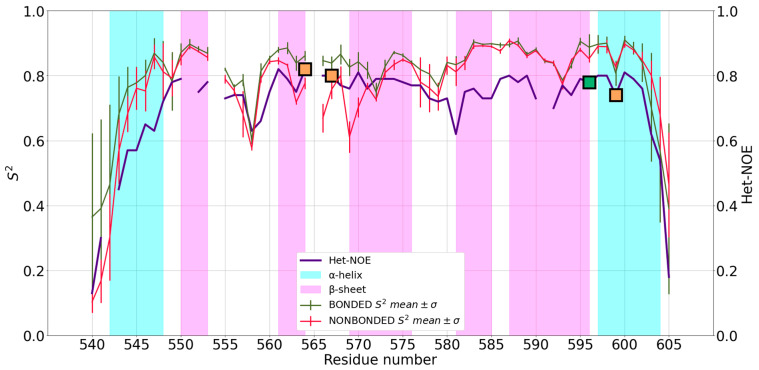
Mean S^2^ of 2NAX and standard deviation (SD) for bonded (red) and nonbonded (green) simulations superimposed to Het-NOE data (purple). Orange squares represent the position in the sequence of zinc(II)-binding Cys residues, whereas the green squares represent zinc(II)-binding His residues.

The first protein in our benchmark set is 2NAX. This structure contains seven β strands, a short N-terminal 3_10_-helix and a longer C-terminal α helix (Figure 1). The residues interacting with the zinc(II) ion are Cys^564^ and Cys^567^, located on the β2β3-hairpin, and His^596^ and Cys^599^ on the C-terminal α helix. Figure 2 shows the averaged MD-computed S^2^ for both FFs superimposed to the Het-NOE data.

The mean S^2^ values computed from the MD runs with the two FFs are almost the same, with a remarkably similar trend over the protein sequence. Moreover, both computed S^2^ display, as expected, high values (>0.7) for regions of secondary structure and lower values for loop regions, identifying a rigid domain in both cases. The ligands participating in metal coordination are encompassed in regions with higher stability than the protein average, whereas the N- and C-termini and loop regions experience significant flexibility. Additionally, with respect to the experimental Het-NOE data, we observed a fully satisfactory agreement, which can be quantitatively expressed by computing the Pearson coefficient. For the 2NAX protein, we obtained a coefficient of 0.82 for the NBFF and of 0.89 for the ZAFF (Table 1). Besides the protein termini, the Het-NOE indicates the presence of a rigid domain, as described above, except around residue 558, which is well captured by our simulations. The simulations with the NBFF display higher than expected flexibility at residues 569–570, which is not observed with ZAFF. This is arguably the largest deviation between the two sets of simulations. The Het-NOE data indicates that the N-terminal helix is looser than the C-terminal; indeed, in the publication reporting the structure, helix α1 was described as tending to partially unfold [23]. We analyzed the trajectories considering and excluding this secondary structure to see whether its presence would influence the prediction of S^2^ values for the whole protein. This was not the case, showing that the local dynamics of helix α1 are effectively decoupled from the rest of the system. Although we inspected several structural factors, namely (i) the distances between the donor atoms, (ii) the distribution of water molecules around the metal site, and (iii) the hydrogen bond patterns, we did not highlight possible causes of the behavior of helix α1.

**Figure 3:**
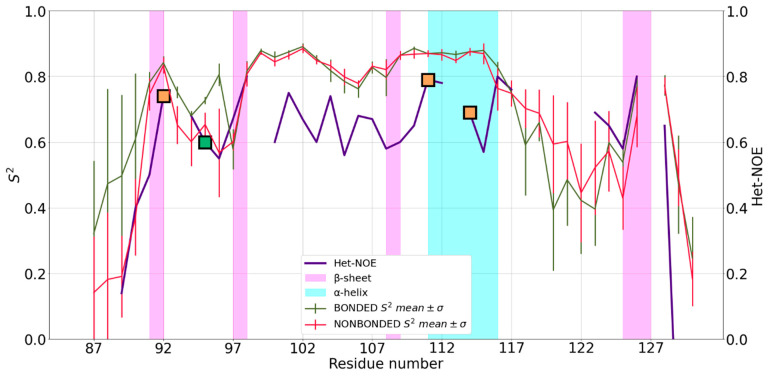
Mean S^2^ of 5JPX and standard deviation (SD) for bonded (red) and non-bonded (green) simulations superimposed to Het-NOE data (purple). Values on y-axis were truncated at 0 because S^2^ has no negative values. Orange squares represent the position in the sequence of zinc(II)-binding Cys residues, whereas the green squares represent zinc(II)-binding His residues.

5JPX presents a ββαβ core domain, with two additional short strands and a disordered N-terminal tail (Figure 1) [24]. The MD-computed S^2^ values for the two FFs are perfectly superimposable in the regions corresponding to secondary structures and show very small differences for loop regions. Both FFs reproduce the local protein dynamics as described by the Het-NOE data (Figure 3), with Pearson coefficients of 0.68 and 0.69 for the NBFF and ZAFF, respectively.

The core encompassing the ligands (Cys^92^, His^95^, Cys^111^ and Cys^114^) is stable during all trajectories, with mean S^2^ values around 0.8, corresponding to well folded secondary structures. The region Arg^118^-Asp^122^ was not characterized experimentally due to signal broadening [24], so no Het-NOE data are available for these residues. The MD simulations provided information about this region indicating that the region 118–122 is highly flexible also on the sub-ns timescale.

**Figure 4:**
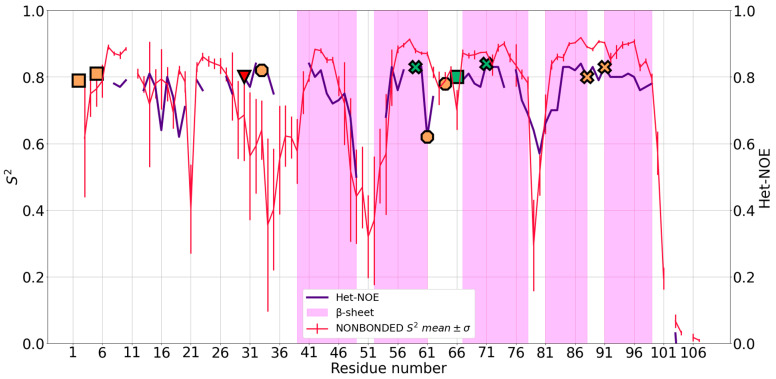
Mean S^2^ of 2JOX and standard deviation (SD) for nonbonded (red) simulations superimposed to Het-NOE data (purple). It was not possible to apply ZAFF to the system. The y-axis was truncated at 0 because S^2^ had no negative values, hence some Het-NOE data for the last protein residues were not visible. Orange markers represent the position in the sequence of zinc(II)-binding Cys residues, whereas the green markers represent zinc(II)-binding His residues. Residues belonging to the same site are represented with the same marker shape (crosses for the mononuclear site; circles for the binuclear site except the bridging Cys^30^, which is represented as a red triangle).

2JOX is composed by an antiparallel β-sheet with five strands, with both sides of the sheet being solvent exposed. The sheet is stabilized by a mononuclear zinc(II) site (His^59^, His^71^, Cys^88^ and Cys^91^) through cross strand interactions. An additional binuclear site (Cys^2^, Cys^5^, Cys^30^, His^66^ for one zinc(II) ion, and Cys^30^, Cys^33^, Cys^61^, Cys^64^ for the other one) holds together the N-terminal region (Figure 1). In this binuclear cluster, Cys^30^ acts as a bridge between the two zinc(II) ions [25]. Due to the specific chemical structure of the binuclear cluster, it was not possible to investigate the system using ZAFF, as it is parametrized mainly for mononuclear sites. At the same time, 2JOX was particularly challenging for the NBFF. Trajectory visualizations and the analysis of donor-metal distances show that during the simulations, the bridging residue acts as a ligand only towards zinc^109^. In other words, the binuclear site splits into two mononuclear sites, with zinc^108^ being coordinated by three residues. The coordination geometry of the latter is kept during all trajectories. Although the computed mean S^2^ (Figure 4) show some discrepancies when superimposed to the experimental Het-NOE data, the overall local protein dynamics is well represented, with a Pearson coefficient of 0.77 (Table 1).

In the N-terminal tail, the loop region 29–39 has a higher predicted flexibility than observed in the experimental data. Notably, for five out of the 11 residues in this region, experimental Het-NOE values are lacking [25]. The mobility enhancement is caused by the displacement of zinc^108^ from zinc^109^, which leads to the rearrangement of this region, resulting in higher solvent exposure and a wider conformational space available. In the β-sheet part of the protein, where the mononuclear site lies, the agreement with the experimental data is excellent. Our simulations are fully consistent with the experimentally observed flexibility in the β1-β2 portion of the sheet, also involving the terminal regions of the two strands. The β-turns between strands β1-β2 and β3-β4 have a higher mobility than the turns between β2-β3 and β4-β5, since they comprise some of the ligands of the zinc(II) ions (Figure 4). The role in metal coordination of these residues restricts their conformational freedom.

**Figure 5:**
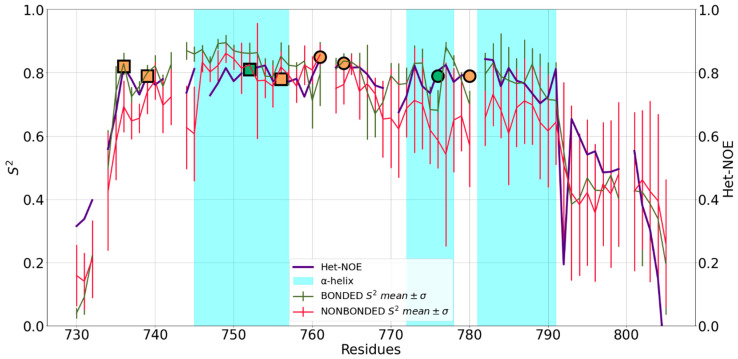
Mean S^2^ of 2L7X and standard deviation (SD) for bonded (red) and nonbonded (green) simulations superimposed to het-NOE data (purple). Values on y-axis were truncated at 0 because S^2^ had no negative values. Orange markers represent the position in the sequence of zinc(II)-binding Cys residues, whereas the green markers represent zinc(II)-binding His residues. Residues belonging to the same site are represented with the same marker shape (squares: ZF1; circles: ZF2).

The 2L7X structure features two zinc fingers, with an additional α3 that packs against the dual zinc finger fold (Figure 1). The N- and C-terminal regions are unstructured and flank the central part of the domain. The first zinc finger (ZF1) bears a zinc(II) ion coordinated by Cys^736^, Cys^739^, His^752^ and Cys^756^, while in ZF2, the coordination is carried out by Cys^761^, Cys^764^, His^776^ and Cys^780^ [26]. The S^2^ values computed from the trajectories with the two FFs are almost superimposable and agree with a rigid and compact structure (Figure 5). ZF1, ZF2 and the linker in between them behave as one entity, whereas the two tails display enhanced flexibility. For both FFs, the predicted dynamics correlate well with the experimental information.

For the 1CHC and 2K9H systems, there are no experimental data for results validation. Thus, we investigated only the relationship between the S^2^ values predicted for the trajectories with the two FFs (Supplementary Figures S7 and S8), as well as with the structural features of the proteins. 1CHC has a split-βαβ topology with an amphipathic α-helix spanning the triple-stranded antiparallel β-sheet [27]. The predicted S^2^ values for both FFs are highly similar and agree with the ZF topology, revealing a stable core with values around 0.8 for the secondary structure elements and for the loop regions harboring the ligands (Supplementary Figure S7). The N- and C-termini flank the compact core and show high flexibility, as expected for unstructured regions. A relevant discrepancy between the behaviors observed with each FF is the enhanced mobility of Cys^32^, which is caused by the lower stability in the NBFF simulations of the secondary structure it belongs to. 2K9H features a novel CCHC dual ZF fold; the ligands of the zinc(II) ions are Cys^548^, Cys^551^, Cys^568^ and His^564^ for the first ZF, and Cys^573^, Cys^576^, His^590^ and Cys^594^ for the second one [28]. This protein has a highly compact structure, which is reflected by the MD-computed mean S^2^ value of each FF. The obtained results are closely superimposable with some discrepancies in the loop region immediately following Cys^576^ (Supplementary Figure S8).

## 3. Discussion

All of the inspected ZFs bear Cys and His ligands coordinating zinc(II) ions in a tetrahedral geometry. The computed results for the two FFs are almost always overlapping and agree with the protein dynamics shown by the Het-NOE data. This is generally true for the regions with limited flexibility (characterized by S^2^ > 0.8 and Het-NOE > 0.7) as well as for the regions with high flexibility outside or within secondary structure elements. To quantify the agreement, we computed the Pearson correlation coefficient between the mean S^2^ of each FF and the corresponding Het-NOE data (Table 1). The Pearson coefficient is an indicator of how accurately each FF represents the experimental trend. The results obtained are satisfactory, with values ranging from 0.68 to 0.89. For the 2JOX protein, the Pearson coefficient is 0.77, suggesting that the NBFF can be useful for systems containing multinuclear sites, for which the traditional ZAFF parametrization is less suitable.

The Pearson coefficients for the ZAFF simulations are marginally better than those obtained with the NBFF, indicating that the two FFs have comparable accuracy. By inspecting this behavior in greater detail, we observed that in some cases, the initial regions of secondary structure elements were not perfectly maintained (information obtained from DSSP analysis, not shown) throughout the trajectories with the NBFF. We speculate that this small destabilization could be due to the electrostatic interaction between the residues forming the secondary structures and the zinc(II) site. In line with this, the mean S^2^ values computed from NBFF trajectories have higher standard deviations than those computed for the ZAFF simulations. This means that the individual trajectories differ more from each other with the former FF than with the latter.

Based on our results, the NBFF and ZAFF are equally reliable for the investigation of zinc(II)-binding proteins, albeit the MD runs with the former have slightly higher standard deviations. In fact, for all the ZFs tested here, both FFs could reproduce properly the local protein dynamics shown by the Het-NOE data. One significant advantage of the NBFF is that it allows dealing with such a diverse protein superfamily as the ZF superfamily. In fact, it can be applied to systems bearing diverse coordination environments in a seamless manner without the need to use a metal center parameter builder, such as MCPB.py [39]. A recent study investigated the ability of different models to reproduce the zinc(II) coordination and the ligand binding in metalloproteins [36]. Among them, the NBFF, used also here, stood up for its great performance in reproducing the geometry and maintaining the correct distances between the ligands and the metal. In contrast, the coordination by His residues was not consistently kept in simulations performed with other non-bonded models [36].

Initially, the NBFF has been tested on the challenging computation of dissociation free energies using alchemical free-energy perturbation for eight zinc(II) proteins with known dissociation constants, featuring very good agreement between computed and experimental dissociation energies [14]. In this contribution, we further validated the NBFF against experimental NMR data probing protein dynamics. An apparent difference among the trajectories obtained with ZAFF and NBFF was that the use of the former resulted in steadier RMSD profiles and more persistent secondary structure elements than for NBFF. This is likely due to the stabilization of the protein topology conferred by the four fixed bonds between the polypeptide chain and each zinc(II) ion. Nevertheless, the S^2^ order parameters calculated from MD trajectories show a highly satisfactory correlation with experimental Het-NOE values for both ZAFF and NBFF, with no significant deviations between the two. Overall, we can conclude that NBFF is well capable of reproducing both energetics parameters and dynamics behavior in zinc(II)-proteins, and thus constitutes a widely adoptable FF for MD simulations of such systems [14,15].

## 4. Methods

### 4.1. Molecular Dynamics Simulations

We performed all MD simulations using the pmemd tool of version 20 of the AMBER software suite. The ff14SB force field (FF) was used to describe the protein chain, whereas the ZAFF [13] and nonbonded FF (NBFF) [14] were applied to the zinc(II) ion and its ligands. For four out of our six selected systems (1CHC, 2JOX, 2L7X and 2K9H), five separate simulations using either the ZAFF or the NBFF were carried out, each of 500 ns duration. Thus, in total, we accumulated 2.5 μs of dynamics with each zinc(II) FF for each system. For the remaining two systems (2NAX and 5JPX), the simulations were 400 ns long, for a total of 2.0 μs of dynamics with each FF for each system with an integration time step of 2 fs; we saved one frame every 5000 steps.

For NMR structures, which are available from the Protein Data Bank as bundles of conformers, we used the first one, since it is usually the one with either the lowest conformational energy or with the best agreement with the NMR restraints [40]. All simulations were performed as follows: the selected protein was embedded in a truncated octahedron box with walls 10 Å away from the solute in each direction. Periodic boundary conditions were applied, and the system was explicitly solvated with TIP3P water model.

The minimization process was performed at 0 K in two steps: (i) minimizing only water molecules and keeping the protein fixed; (ii) minimizing the whole system. For this process, a combination of Steepest Descendent and Conjugated Gradient algorithms was exploited. Subsequently, the system was heated to 300 K at constant volume using the weak-coupling algorithm. The system was then equilibrated at constant pressure and temperature in NPT ensemble using a Berendsen barostat. During the heating procedures, bond constraints were imposed on X-H bonds using the SHAKE algorithm, omitting the force evaluation of bonds containing hydrogen. The latter protocol was applied also for the MD production runs, with an increased number of integration steps. The input files used for the simulations are provided as Supplementary Materials, using the example of 2L7X.

The root mean square deviation (RMSD) is a measure of the similarity between two superimposed 3D structures, defined by the formula:

$$\mathrm{RMSD}=\sqrt{\frac{1}{n}\overset{n}{\underset{i=1}{\sum }}d_{i}^{2}}$$

where the averaging is performed over *n* pairs of equivalent atoms, and *d_i_* is the distance between two atoms each belonging to a conformation [41]. We computed RMSD values over the backbone atoms to keep track of how the protein behaved during the simulations. RMSD data were computed relative to the equilibrated structure using cpptraj [42]. The latter software was exploited to compute the distances between the atom participating in the coordination and the zinc(II) ion. The content of secondary structures was computed using DSSP. Its dictionary contains eight classes of possible structures: random coil, parallel beta-sheet, antiparallel beta-sheet, 3–10 helix, alpha-helix, Pi (3–14) helix, turn and bend [43].

### 4.2. Order Parameters

The order parameter (S^2^) describes the magnitude of the angular fluctuation of a chemical bond vector such as the N-H bond in proteins, reflecting the flexibility of the polypeptide at those sites with respect to the overall protein frame [31,34]. For S^2^ = 0, the internal motion spans all possible orientations, whereas S^2^ = 1 corresponds to complete rigidity [34,35]. Using our simulations, we computed the S^2^ values for the backbone N-H vectors of all the investigated systems with the isotropic reorientational eigenmode dynamics (iRED) method [29]. The final order parameters with their respective standard deviations (SDs) were obtained by averaging the results for the independent simulations run with each FF. For each protein, we compared the averaged S^2^ obtained with the two zinc(II) FFs to assess their similarity. In addition, to evaluate how well the experimental data were reproduced by the tested FFs, we computed the Pearson correlation coefficient with respect to the Het-NOE data using the pandas library [44,45].

VMD was used for the inspection of trajectories, and Pymol for the visualization of extracted frames [46,47].

## Figures and Tables

**Table 1:** Pearson coefficients computed for each zinc(II) FF with respect to the Het-NOE data. For 2JOX, it was not possible to apply the ZAFF. 1CHC and 2K9H are not reported since there are no experimental data available.

Zinc-Fingers	Pearson Coefficient for NBFF	Pearson Coefficient for ZAFF
2NAX	0.82	0.89
5JPX	0.68	0.69
2JOX	0.77	n.a.
2L7X	0.79	0.84

## Supplementary Materials

The following supporting information can be downloaded at: https://www.mdpi.com/article/10.3390/ijms24065440/s1.
