# Supplementary material for: A Comparison of Bonded and Nonbonded Zinc(II) Force Fields with NMR Data
Source: Int J Mol Sci. 2023 Mar 13;24(6):5440. doi: 10.3390/ijms24065440 (PMC10055966; doi:10.3390/ijms24065440)
Supplement: Supplementary file 1 [file ijms-24-05440-s001.zip › simulations_setting.pdf]

The following input files were used for 2L7X and are reported here as an example of the protocol used to run the MD simulations for all systems.

NONBONDED SIMULATIONS input files

min.in

```
Minimization with Cartesian restraints for the solute
 &cntrl
 imin=1, maxcyc=200,
 ntpr=5,
 ntr=1,restraint_wt=10,restraintmask='1-77'
/
```

min2.in

```
Minimization with Cartesian
 &cntrl
 imin=1, maxcyc=500,
 ntpr=5,
 ntr=0
/
```

heat1.in

```
Heating up the system equilibration stage 1
 &cntrl
  nstlim=50000, dt=0.002, ntx=1, irest=0, ntpr=500, ntwr=5000, ntwx=50,
  tempi =100.0, temp0=300.0, ntt=1, tautp=2.0, ig=-1,

  ntb=1, ntp=0,

  ntc=2, ntf=2,

  nrespa=2,
&end
```

heat2.in

```
Constant pressure constant temperature equilibration stage 2
 &cntrl
  nstlim=5000, dt=0.002, ntx=5, irest=1, ntpr=500, ntwr=5000, ntwx=5000,
  temp0=300.0, ntt=1, tautp=2.0,

  ntb=2, ntp=1,

  ntc=2, ntf=2,
```

```
  nrespa=1,
&end
```

dina.in

```
Constant pressure constant temperature production run
 &cntrl
  nstlim=50000000, dt=0.002, ntx=5, irest=1, ntpr=500, ntwr=5000, ntwx=5000,
  temp0=300.0, ntt=1, tautp=2.0,

  ntb=2, ntp=1,

  ntc=2, ntf=2,

  nrespa=1,
&end
```

dina2.in

```
Constant pressure constant temperature production run
 &cntrl
  nstlim=50000000, dt=0.002, ntx=5, irest=1, ntpr=5000, ntwr=5000, ntwx=5000,
  temp0=300.0, ntt=1, tautp=2.0,

  ntb=2, ntp=1,

  ntc=2, ntf=2,

  nrespa=1,
&end
```

dina3.in

```
Constant pressure constant temperature production run
 &cntrl
  nstlim=50000000, dt=0.002, ntx=5, irest=1, ntpr=5000, ntwr=5000, ntwx=5000,
  temp0=300.0, ntt=1, tautp=2.0,

  ntb=2, ntp=1,

  ntc=2, ntf=2,
```

```
  nrespa=1,
&end
```

dina4.in

```
Constant pressure constant temperature production run
 &cntrl
  nstlim=50000000, dt=0.002, ntx=5, irest=1, ntpr=5000, ntwr=5000, ntwx=5000,
  temp0=300.0, ntt=1, tautp=2.0,

  ntb=2, ntp=1,

  ntc=2, ntf=2,

  nrespa=1,
&end
```

dina5.in

```
Constant pressure constant temperature production run
 &cntrl
  nstlim=50000000, dt=0.002, ntx=5, irest=1, ntpr=5000, ntwr=5000, ntwx=5000,
  temp0=300.0, ntt=1, tautp=2.0,

  ntb=2, ntp=1,

  ntc=2, ntf=2,

  nrespa=1,
&end
```

BONDED SIMULATIONS input files

min.in

```
&cntrl
 imin=1, maxcyc=200,
 ntpr=5,
 ntr=1,restraint_wt=10,restraintmask=':1-77'
/
```

min2.in

```
Minimization with Cartesian
 &cntrl
 imin=1, maxcyc=500,
 ntpr=5,
 ntr=0
/
```

heat1.in

```
Heating up the system equilibration stage 1
 &cntrl
  nstlim=50000, dt=0.002, ntx=1, irest=0, ntpr=500, ntwr=5000, ntwx=50,
  tempi =100.0, temp0=300.0, ntt=1, tautp=2.0, ig=-1,

  ntb=1, ntp=0,

  ntc=2, ntf=2,

  nrespa=2,
&end
```

heat2.in

```
Constant pressure constant temperature equilibration stage 2
 &cntrl
  nstlim=5000, dt=0.002, ntx=5, irest=1, ntpr=500, ntwr=5000, ntwx=5000,
  temp0=300.0, ntt=1, tautp=2.0,

  ntb=2, ntp=1,

  ntc=2, ntf=2,

  nrespa=1,
&end
```

dina.in

```
Constant pressure constant temperature production run
 &cntrl
  nstlim=50000000, dt=0.002, ntx=5, irest=1, ntpr=500, ntwr=50000, ntwx=5000,
  temp0=300.0, ntt=1, tautp=2.0,

  ntb=2, ntp=1,

  ntc=2, ntf=2,
```

```
  nrespa=1,
&end
```

dina2.in

```
Constant pressure constant temperature production run
 &cntrl
  nstlim=50000000, dt=0.002, ntx=5, irest=1, ntpr=500, ntwr=50000, ntwx=5000,
  temp0=300.0, ntt=1, tautp=2.0,

  ntb=2, ntp=1,

  ntc=2, ntf=2,

  nrespa=1,
&end
```

dina3.in

```
Constant pressure constant temperature production run
 &cntrl
  nstlim=50000000, dt=0.002, ntx=5, irest=1, ntpr=500, ntwr=50000, ntwx=5000,
  temp0=300.0, ntt=1, tautp=2.0,

  ntb=2, ntp=1,

  ntc=2, ntf=2,

  nrespa=1,
&end
```

dina4.in

```
Constant pressure constant temperature production run
 &cntrl
  nstlim=50000000, dt=0.002, ntx=5, irest=1, ntpr=500, ntwr=50000, ntwx=5000,
  temp0=300.0, ntt=1, tautp=2.0,

  ntb=2, ntp=1,

  ntc=2, ntf=2,

  nrespa=1,
&end
```

dina5.in

```
Constant pressure constant temperature production run
 &cntrl
  nstlim=50000000, dt=0.002, ntx=5, irest=1, ntpr=500, ntwr=50000, ntwx=5000,
  temp0=300.0, ntt=1, tautp=2.0,

  ntb=2, ntp=1,

  ntc=2, ntf=2,

  nrespa=1,
&end
```
