# Supplementary material for: A Comparison of Bonded and Nonbonded Zinc(II) Force Fields with NMR Data
Source: Int J Mol Sci. 2023 Mar 13;24(6):5440. doi: 10.3390/ijms24065440 (PMC10055966; doi:10.3390/ijms24065440)
Supplement: Supplementary file 1 [file ijms-24-05440-s001.zip › supplementary figures.pdf]

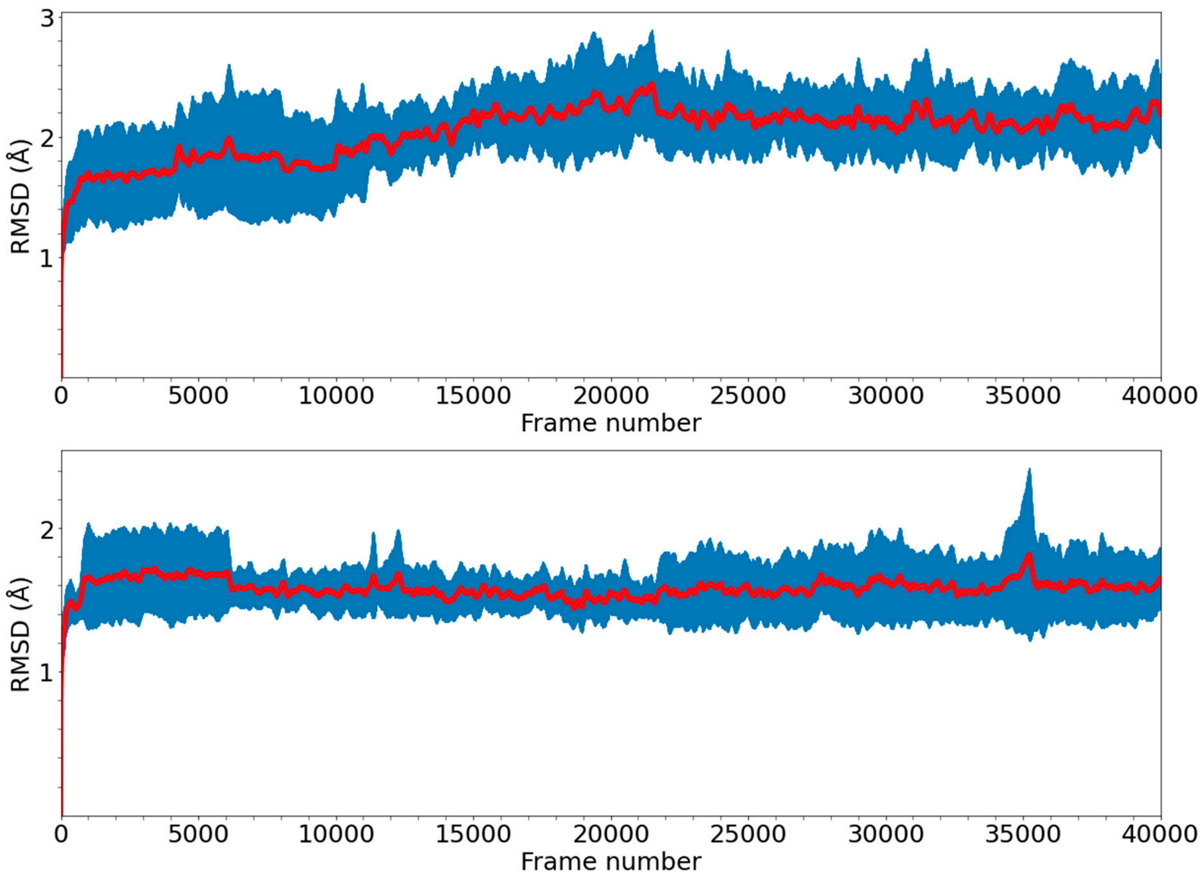

Figure S1. Smoothed mean RMSD for 2NAX computed for the NBFF (upper panel) and ZAFF (lower panel).

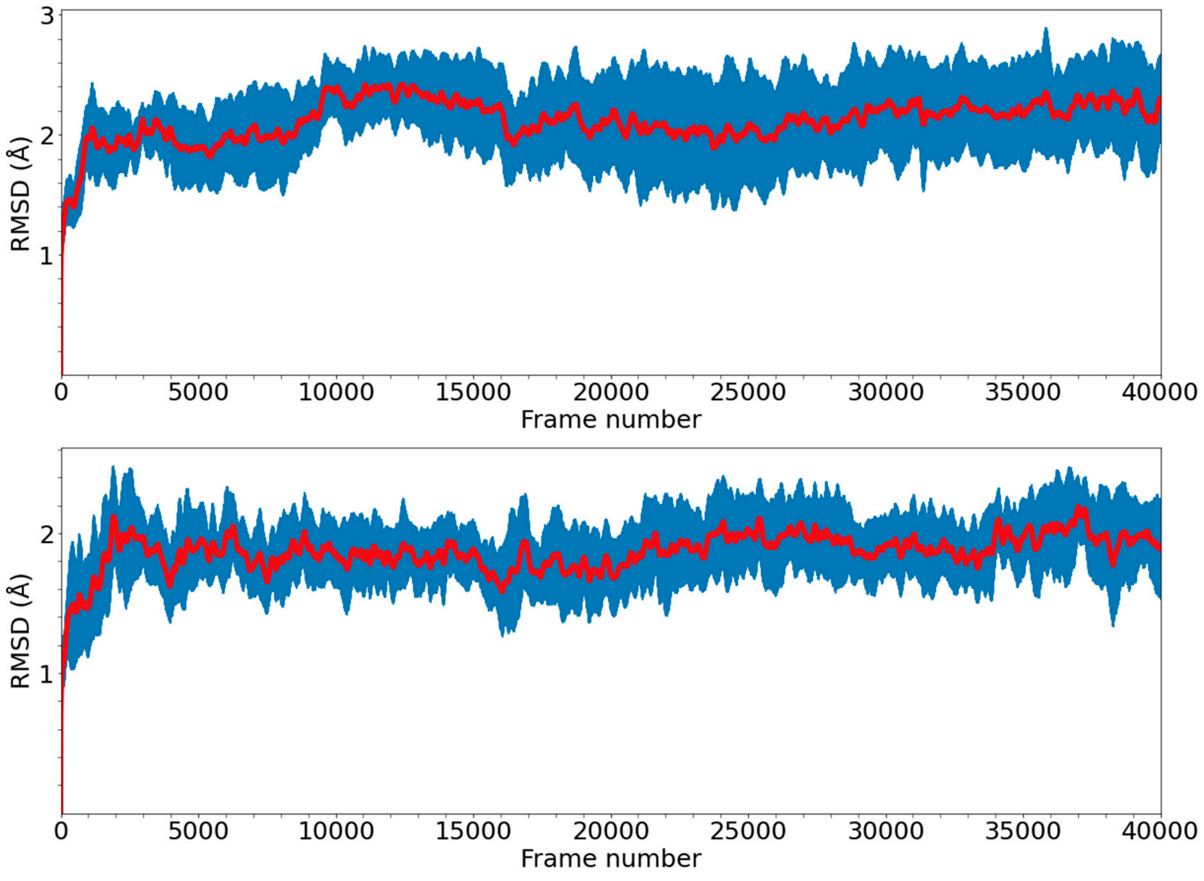

Figure S2. Smoothed mean RMSD for 5JPX computed for the NBFF (upper panel) and ZAFF (lower panel).

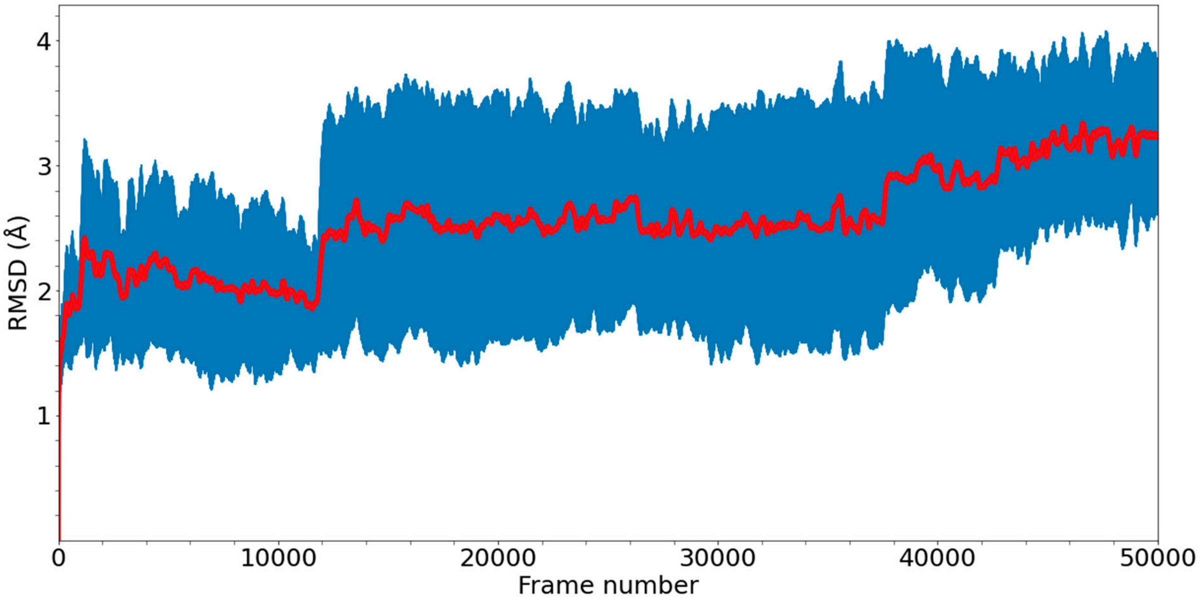

Figure S3. Smoothed mean RMSD for 2JOX computed for the NBFF. Simulations with ZAFF were not performed since it is not parametrized for binuclear sites.

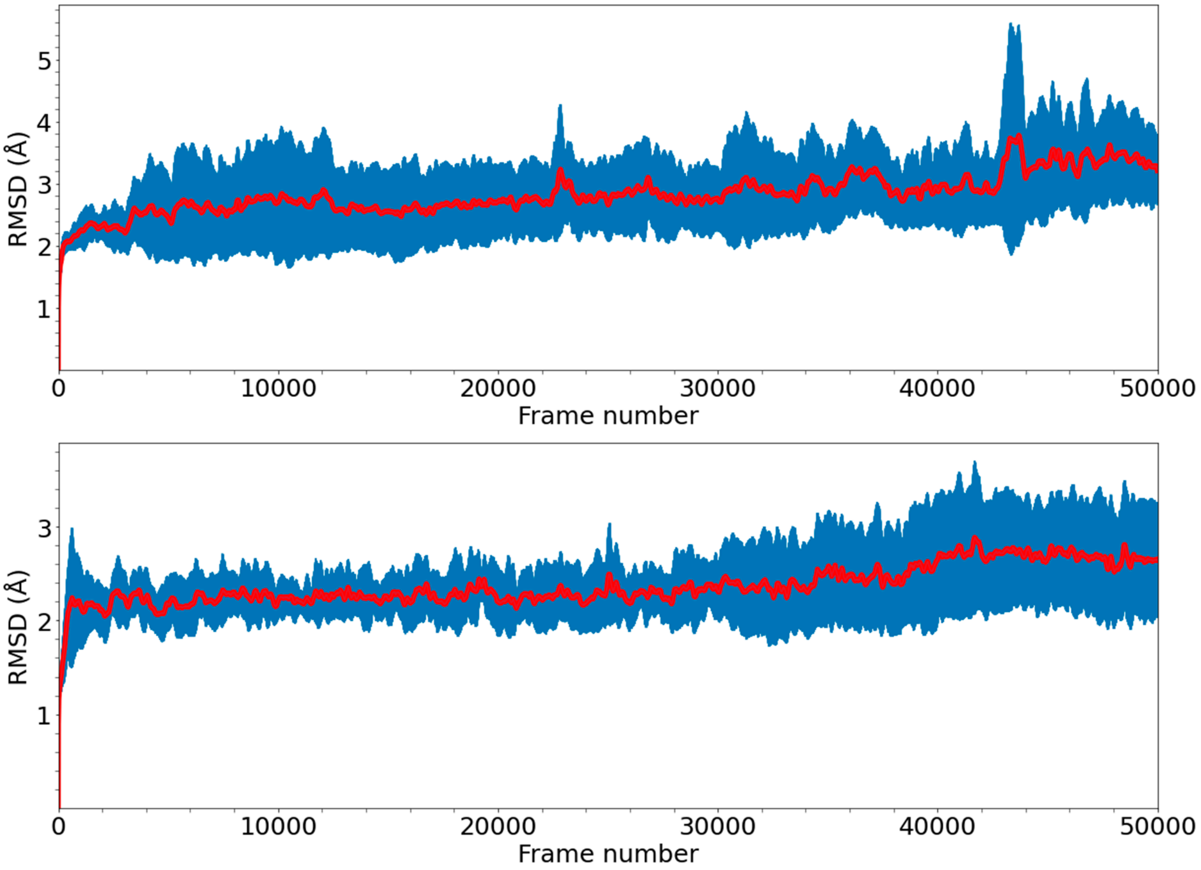

Figure S4. Smoothed mean RMSD for 2L7X computed for the NBFF (upper panel) and ZAFF (lower panel).

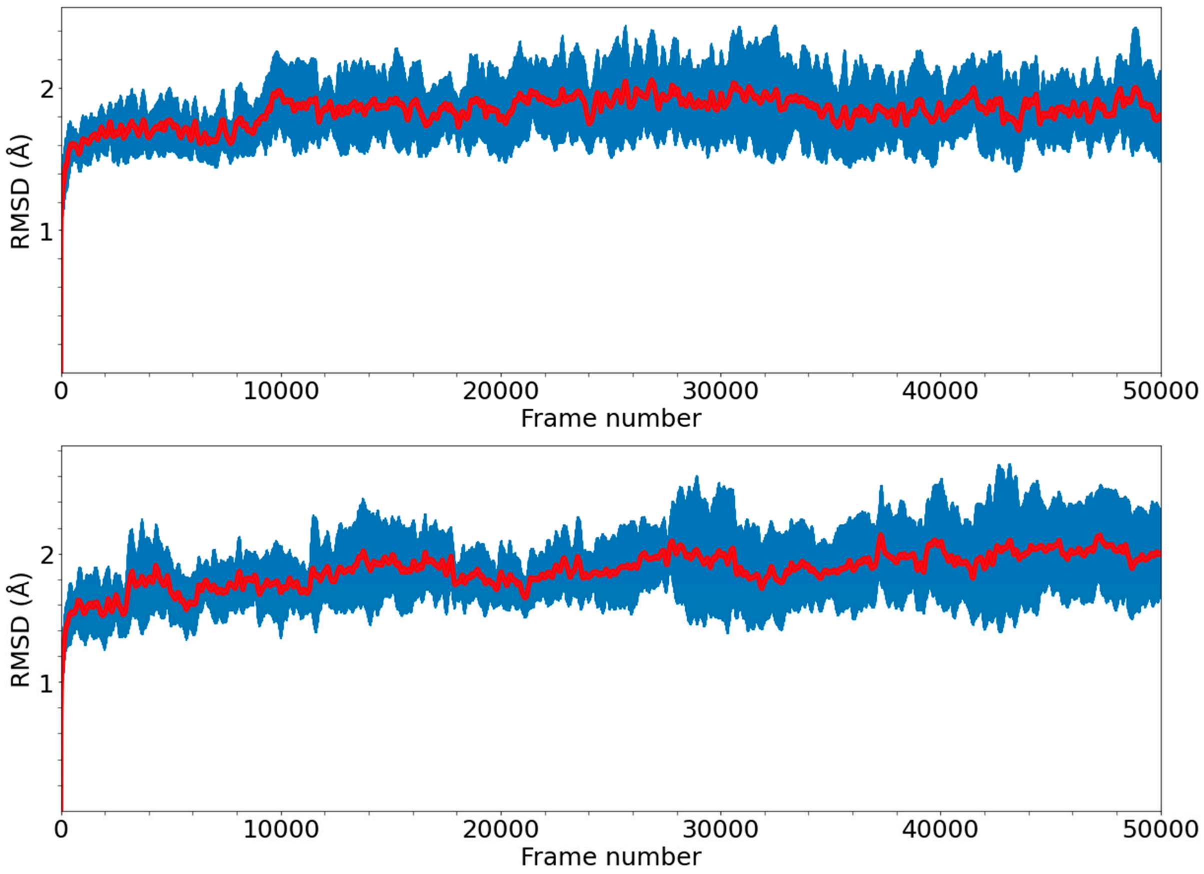

Figure S5. Smoothed mean RMSD for 1CHC computed for the NBFF (upper panel) and ZAFF (lower panel).

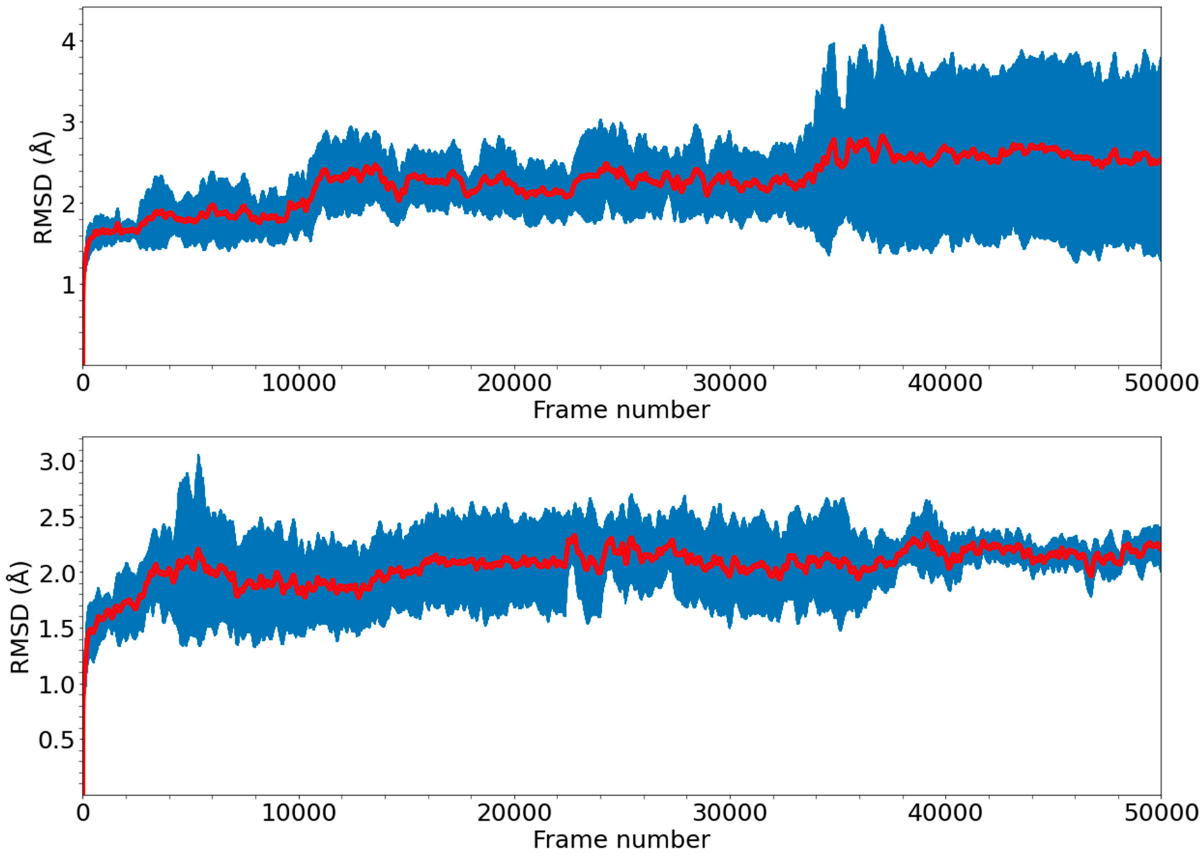

Figure S6. Smoothed mean RMSD for 2K9H computed for the NBFF (upper panel) and ZAFF (lower panel).

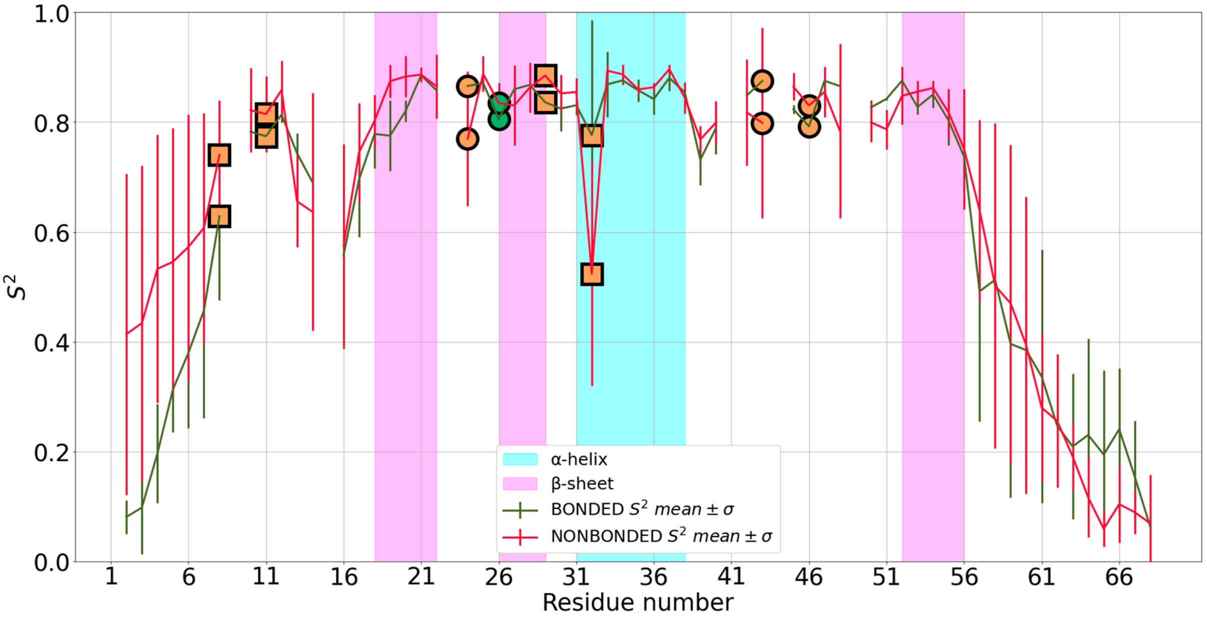

Figure S7. Mean S^2 of 1CHC and standard deviation (SD) for bonded (red) and nonbonded (green) superimposed. Orange markers represent the position in the sequence of zinc(II)-binding Cys residues, whereas the green markers represent zinc(II)-binding His residues. Residues belonging to the same site are represented with the same marker shape.

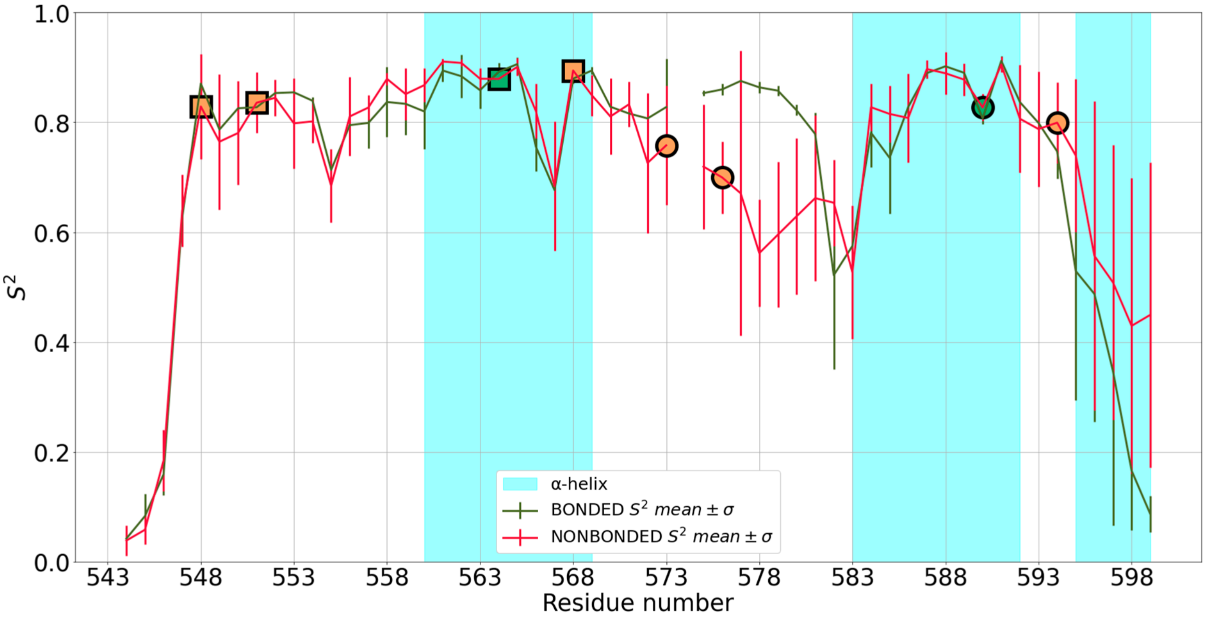

Figure S8. Mean S^2 of 2K9H and standard deviation (SD) for bonded (red) and nonbonded (green) superimposed. Orange markers represent the position in the sequence of zinc(II)-binding Cys residues, whereas the green markers represent zinc(II)-binding His residues. Residues belonging to the same site are represented with the same marker shape.
